# The Practice of Fast-Track Liver Transplant Anesthesia

**DOI:** 10.3390/jcm12103531

**Published:** 2023-05-18

**Authors:** Stephen Aniskevich, Courtney L. Scott, Beth L. Ladlie

**Affiliations:** Department of Anesthesiology, Mayo Clinic Florida, Jacksonville, FL 32224, USA; scott.courtney@mayo.edu (C.L.S.); ladlie.beth@mayo.edu (B.L.L.)

**Keywords:** early extubation, fast-track, liver transplantation, anesthesia

## Abstract

Prior to the 1990s, prolonged postoperative intubation and admission to the intensive care unit was considered the standard of care following liver transplantation. Advocates of this practice speculated that this time allowed patients to recover from the stress of major surgery and allowed their clinicians to optimize the recipients’ hemodynamics. As evidence in the cardiac surgical literature on the feasibility of early extubation grew, clinicians began applying these principles to liver transplant recipients. Further, some centers also began challenging the dogma that patients need to be cared for in the intensive care unit following liver transplantation and instead transferred patients to the floor or stepdown units immediately following surgery, a technique known as “fast-track” liver transplantation. This article aims to provide a history of early extubation for liver transplant recipients and offer practical advice on how to select patients that may be able to bypass the intensive care unit and be recovered in a non-traditional manner.

## 1. Introduction

Surgical and anesthetic management for liver transplantation has evolved since the first liver transplant was performed by Dr. Starzl in 1963. Transplant centers have started pushing boundaries to improve the availability of organs for their patients on the waitlist. These include utilizing machine perfusion and using organs historically considered to be marginal grafts, such as older donation after cardiac death (DCD) donors and donors with hepatitis C [1]. Additionally, surgical techniques and experience have improved with surgeries now taking less time and resulting in less hemodynamic instability [2]. Concurrent with this, the practice of transplant anesthesia has also evolved. Transplant anesthesiologists now play an integral role on patient selection committees at many centers, and improvements in intraoperative monitoring allow anesthesiologists to care for sicker patients that would have historically been denied transplant [3]. Now some centers are challenging the dogma that patients need to be cared for in the intensive care unit (ICU) following liver transplantation and are instead transferring patients to the floor or stepdown units immediately following surgery, a technique known as “fast-track” liver transplantation. This article aims to provide a history of early extubation for liver transplant recipients and offer practical advice on how to select patients that may be able to bypass the ICU and be recovered in a non-traditional manner.

## 2. History

The concept of early extubation for liver transplant patients has its roots in the cardiac surgery literature. Prior to the 1970s, intensivists and anesthesiologists held to the belief that prolonged ventilation for at least 24 h after cardiac surgery ensured adequate oxygenation, prevented atelectasis, and decreased the metabolic requirements associated with pain-induced tachypnea [4,5,6]. This viewpoint started to change in the 1970s when several papers were published showing early extubation was feasible and not associated with increased mortality. In 1974, Midell showed that early extubation was possible in patients undergoing cardiac valve surgery [7]. Klineberg demonstrated that early extubation following coronary artery bypass was safe if specific clinical criteria were met and resulted in significantly shorter admissions and ICU stays in the early extubation group [8]. Several years later, Prakash et al. expanded on this by describing early extubation following cardiac surgery [9]. His team found that, with a careful clinical and physiological evaluation, 87% of patients undergoing open-heart surgery could be extubated within 3 h of surgery. Only five patients out of the initial 123 early extubation patients needed reintubation, three of these due to nonpulmonary complications.

As comfort with early extubation following cardiac surgeries grew, transplant anesthesiologists began applying these criteria to liver recipients. Early proponents suggested that early extubation may help liver graft function by decreasing hepatic arterial and venous congestion associated with positive pressure ventilation. Most of this early evidence was supported by animal studies; however, PEEP-induced liver dysfunction has not been substantiated in human studies [10,11,12,13,14,15]. More recently, Yuan, et al., examined 10,517 liver transplant patients and found that prolonged intubation greater than 96 h was associated with a three-fold increased mortality risk in the first year following transplant [16]. While a definitive association with ventilation and mortality was not discussed, patients with multiple comorbidities and poor graft function likely contributed to the need for prolonged intubation and poor outcomes. The first article describing early extubation following liver transplantation was published by Roissant. This team found that the judicious administration of fluid to only when there was a decrease in the cardiac filling pressures allowed 5 of 36 patients to be immediately extubated after surgery [17]. The remaining 31 patients were extubated within six hours of surgery. Mandell et al. published the first multicenter study evaluating early extubation in 1997 [18]. This study utilized two separate methodologies to determine if the patient was a candidate for extubation. At the University of Colorado (UC), patients who were extubated within 12 h following liver transplant were evaluated, and extubation criteria were derived and subsequently applied prospectively. At the University of San Francisco (UCSF), patients were extubated based on clinical judgement, and then these patients were retrospectively studied to evaluate for criteria that lead to successful extubation. In total, 16/67 patients at UC and 25/106 patients at UCSF were successfully extubated. Two patients in the UCSF group had to be reintubated due to respiratory issues. The authors concluded that early extubation was feasible using either conservative criteria or clinical judgement. More importantly, early extubation resulted in a decreased need for ICU care which translated to a significant cost savings. Neelakanta compared 18 patients who were successfully extubated immediately after surgery to 17 patients who were left intubated [19]. This group found that early extubation was safe, however, contrary to Mandell, it did not result in a reduction in ICU or hospital stays.

The new millennium brought an increase in the number of articles describing early extubation. Biancofiore published two studies looking at early extubation over both a two-year and five-year period using standard extubation criteria [20,21]. The authors concluded in both studies that extubation can be safely performed based on clinical judgement and without the use of predefined criteria. In a small study, Cammu et al. found that living-donor liver recipients could also be successfully extubated if specific preoperative and intraoperative criteria were met [22]. Glanemann retrospectively reviewed extubation practices in 546 patients. Of these, 18.7% were successfully extubated in the operating room with a reintubation rate of 8.8%. Seventy percent required intubation for less than 5 h postoperatively, and 11% required prolonged ventilation for more than 24 h. They concluded that the risk of reintubation for the immediate extubation group was not increased compared to patients who were extubated later [23]. This conclusion has been supported by many studies since these initial reports. In general, most authors used predefined criteria encompassing transfusion amounts, baseline comorbidities (especially the absence of encephalopathy), and lab values to define who would likely be successfully extubated following surgery [22,24,25,26,27,28,29,30]. Even with protocols using defined physiological and lab criteria, individual provider experience was found to be a major contributor to increased rates of early extubation, with increased success rates as providers became comfortable with the idea of early extubation [20,31,32]. While early extubation has been shown to be safe in living-donor liver transplant recipients [33,34], there are no studies evaluating “fast-track” anesthesia in this population. Presumably this is due to the small number of programs with fast-track processes in place.

As the literature surrounding early extubation grew, it became apparent that a wide range of variables could be used to predict early extubation success. In an attempt to standardize these criteria and give guidance, several groups devised scoring systems utilizing preoperative and intraoperative measurements. Skurzak et al. was among the first to devise a scoring system utilizing major and minor criteria [35]. Termed the SORELT (Safe Operating Room Extubation after Liver Transplantation) score, these authors defined major criteria as (1) more than 7 units of packed cells transfused and (2) a lactate more than 3.4 mmol/L. The three minor criteria were if the patient was admitted at the time of surgery, a surgery lasting more than 5 h, and the need for vasoactive infusions at the end of surgery. Patients with either both major, all 3 minor, or 1 major and 2 minor criteria were unlikely to be good candidates for early extubation. Bulatao et al. expanded on this and devised a scoring system that not only predicted early extubation but also helped to predict which patients could bypass a postoperative ICU admission and be directly admitted to the surgical ward [36]. Their probability score utilized 9 variables to generate a numerical score that determined successful “fast-track” candidates with a predictive value of 93.3% in patients associated with higher scores. This scoring system was subsequently independently applied to a cohort at Toronto General Hospital [37]. While the Toronto team felt the scoring system had utility in predicting fast-track probability, they felt regional population and practice differences needed to be considered and may affect the accuracy of the model. More recently, a group in Thailand devised a simplified scoring system based on a single-center retrospective review of successful extubations [32]. This team assigned two points for a MELD score less than 25, 1 point for <1600 cc of packed red blood cell transfusions, and 2 points for no postoperative inotrope support. They found a score of 5 had a sensitivity of 61%, specificity of 80.25%, and a diagnostic accuracy of 74.06% for successful extubation.

In addition to avoiding the medical complications of prolonged ventilation, early extubation and bypassing the ICU can result in significant cost savings. In 1997, Mandell showed that early extubation reduced the time in the ICU by an average of 15 h which resulted in fewer chest radiograms, blood gases, and staffing costs [18]. In total, this translated to a cost savings of $2709 per patient. Taner et al. also found a significant cost savings in patients who bypassed the ICU following early extubation [38]. Similar to Mandell, they found a reduction in the number of laboratory and radiographic studies, as well as a reduction in room charges. Loh et al. examined the financial impact of the fast-track liver transplant practice at Mayo Clinic Florida [39]. They found that bypassing the ICU postoperatively likely resulted in a reduction in the length of stay by 2.5–3.2 days. The 204 patients fast tracked during the 3-year study period translated to a cost savings of $2–2.6 million for the institution. If the cost savings incurred by this practice was extrapolated to all transplant programs in the United States, the saving to the US health care system would approximate $39–50 million per year. Early extubation and bypassing the ICU does come with some risk. Wu et al. evaluated 11 studies published before 2012 and found that reintubation rates varied from 1.3–35.6% with reoperation playing a significant role in most studies [40]. Most commonly, the need for reintubation was related to surgical complications, respiratory insufficiency, and cerebral events. Wu also suggested that physiotherapy or noninvasive positive pressure ventilation may help prevent reintubation for cases of mild respiratory insufficiency in the recovery period. This assertation has been supported by several studies in both the adult and pediatric liver transplant literature [21,25,41]. Outside of potential benefits to the pulmonary and hepatic system, Bhatia suggested early extubation may prevent acute kidney injury following liver transplantation. In their study, Bhatia found that decreasing the need for vasopressors to offset the hypotension associated with sedative use resulted in a trend toward less renal replacement therapy in the fast-track group [42]. They concluded that early extubation may convey a clinical benefit to the patient in addition to a resource benefit to the institution.

## 3. Anesthetic Management

The goal of anesthesia management for early extubation focuses around maintaining good postoperative analgesia with minimal respiratory depression. Several authors have recommended enhanced recovery pathways for liver transplantation utilizing various strategies to manage pain and aid with recovery in the perioperative period [43,44,45,46]. Most of the initial studies on early extubation utilized a balanced anesthetic approach, with volatile anesthetics supplemented with short acting narcotics such as fentanyl or remifentanil [17,18,19,20,23,35,47]. Neuromuscular blockade with cisatracurium, vecuronium, or pancuronium was commonly used in these early studies. Benzoisoquinolones, such as cisatracurium, may be the neuromuscular blockers of choice due to their extrahepatic metabolism and decreased risk of prolonged paralysis in cases of delayed graft function. With the advent of sugammadex, routine use of rocuronium and vecuronium can also be considered. While studies examining sugammadex in liver transplants are limited, it appears to be effective and safe in patients with liver dysfunction undergoing hepatic resections [48,49]. Deana and colleagues compared sugammadex reversal of continuous rocuronium infusions in liver transplantation versus neostigmine [50]. They found that reversal with sugammadex was safe and faster than with neostigmine; however, the recovery time in the transplant group was considerably longer than has been described in other surgical settings. In this study, up to 33% of patients needed more than 10 min (mean of 9.4 min) to attain a train of four ratio >0.9 compared to 34.6 min in the neostigmine group. Propofol infusions can also be utilized during liver transplant; however, providers should be aware that elevated concentrations have been described during the anhepatic phase due to altered metabolism [51,52]. It is probably prudent to utilize a bispectral index to gauge the depth of anesthesia and prevent delayed emergence when using propofol infusions.

Judicious use of opioids should be considered to prevent postoperative respiratory depression and facilitate early extubation. Liver transplant recipients historically have been assumed to have a lower opioid requirement compared to patients undergoing other hepatobiliary procedures [53,54]. The exact etiology for this finding is not well understood; however, Donovan et al. postulated that metenkephalins may play a role [55]. In their study, 3 neuropeptides known to modulate pain were measured (metenkephalin, beta-endorphin, and substance P) in liver transplant patients and in 9 control patients undergoing liver resection. Of the 3 neuropeptides, only metenkephalin was significantly elevated preoperatively, intraoperatively, and for the first three days following transplantation. The authors felt this was likely due to a combination of decreased metabolism and increased production of “cryptic” prohormones, such as preproenkephalin. To the contrary, Milan argues that everyday practice does not always support this belief and that the size and location of the incision, the loss of intraoperatively administered pain medications with massive blood loss, and increased metabolism of the newly functioning graft may lead physicians to undertreat pain postoperatively [56].

The use of regional nerve blocks in liver recipients may help to alleviate pain and avoid opioid-induced respiratory depression. Two European studies have evaluated the use of thoracic epidural analgesia (TEA) to control pain in a small subset of liver recipients with normal preoperative coagulation prior to placement. A team at the Medical University of Warsaw reported the use of TEA on 67/279 liver recipients with an INR < 1.5, APTT < 45 s, and platelets > 70 × 10(9)/L [57]. The use of thromboelastography was also utilized when available. Of the 67 patients, only 5 had unsatisfactory pain control, and more importantly, there were no complications associated with TEA use. Hausken et al. described the use of TEA in 327 liver recipients. They also required patients to have normal coagulation profiles preoperatively, defined as an INR < 1.5, and platelet counts greater than 100 × 10(9)/L. They found similar pain control without any serious bleeding or persistent neurological complications [58]. Subcostal transversus abdominis plane (TAP) block has also been described as a potential adjunct to help with pain control [59,60]. The benefit of this approach is that it does not require a patient to have a normal coagulation profile. Milan evaluated bilateral subcostal TAP blocks using levobupivacaine and found they resulted in a significant decrease in morphine consumption compared to controls [59]. Assefi found that subcostal TAP blocks using ropivacaine reduced morphine consumption but did not result in a difference in pain scores [61]. Providers should be cautious with TAP placement as accidental liver puncture has been described in lower abdominal surgeries and may be a risk in patients with muscle wasting receiving a slightly larger graft [59,62,63]. External oblique intercostal nerve blocks hold promise as an alternative to provide upper abdominal analgesia; however, studies evaluating their efficacy in liver transplant recipients is lacking [64,65,66,67].

## 4. Fast-Track Anesthesia at Mayo Clinic Florida

**Figure 1:**
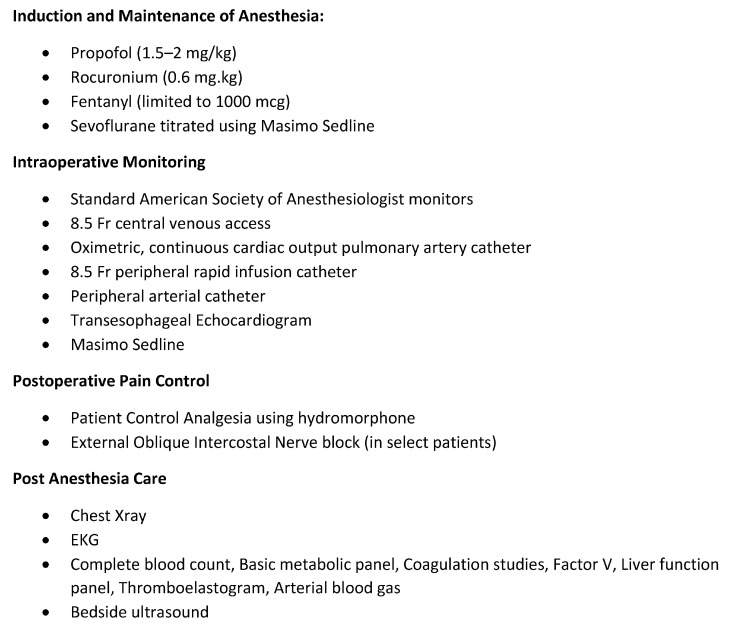
Mayo Clinic Florida Anesthesia Protocol for Fast Track Liver Transplant.

**Figure 2:**
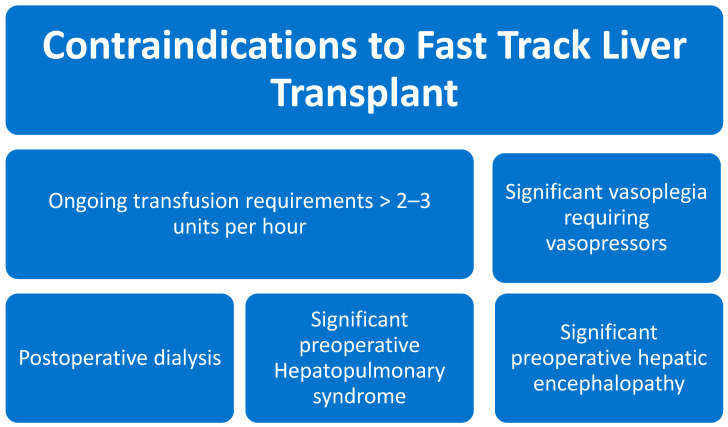
Factors that preclude fast tracking at our institution.

We have been practicing fast-track anesthesia at Mayo Clinic for 20 years with approximately 60% of our liver recipients extubated in the operating room and transferred to the surgical ward following a short stay in the recovery room. We have previously described our anesthetic approach [47] (Figure 1). The decision to fast track our patients is at the discretion of the transplant anesthesiologist in consultation with the surgical team. In general, we typically assess the need for postoperative transfusion, hemostasis, and comorbidities when determining where to recover a patient. Factors that preclude fast tracking at our institution include an ongoing transfusion requirement of >2–3 units per hour or significant coagulopathy (INR > 2.5), need for vasopressor support or postoperative dialysis, significant hepatopulmonary/portal pulmonary syndrome, or significant preoperative encephalopathy (Figure 2). While massive transfusion cases or redo transplants do not necessarily require an ICU stay, we err on the side of caution if there are concerns of continued oozing of raw surfaces or transfusion-related lung injury. In the recovery room, we check baseline lab studies, treat pain, and monitor the patient for a minimum of 1 h to ensure they are stable prior to discharge. Our transplant floor effectively functions as a stepdown unit where postoperative liver recipients receive 1:1 nursing care and continuous monitoring for the first 24 h. Additionally, we have a midlevel provider available in-house with an intensivist available as backup for emergencies. There is a transplant hepatologist and a surgeon on call 24/7 for consultation and management of non-emergent medical issues.

Transitioning from the standard postoperative ICU admission to a fast-track program can be a difficult process and requires buy-in from multiple levels of providers across several specialties to alleviate safety and cost concerns. It is important to have physicians, nursing leadership, and administration involved early with the expectation that there will be a significant learning curve early on. Additionally, there can be a significant initial capital investment needed to ensure the receiving floor can staff and monitor these patients adequately, and this may not be recuperated quickly for smaller volume programs.

## 5. Conclusions

Early extubation following liver transplantation is now practiced at many centers around the world and has been shown to be safe when based on either predefined criteria or clinical judgement. Expanding on this, centers are now challenging the dogma that liver transplant patients need to be recovered in the intensive care setting, and some programs have developed care systems that allow select patients to bypass the ICU and go directly to the surgical ward. When performed properly, this practice is safe and has a clinical benefit to the patient, as well as a cost-saving benefit to the institution and health system. Furthermore, by decreasing the need for ICU admissions, fast tracking liver transplant recipients may promote the growth of transplant volume in areas hindered by shortages in critical care beds and staff and result in cost saving to the health system.
